# (*Z*)-4-{1-[(2-Hy­droxy­ethyl)­amino]­ethyl­idene}-3-methyl-1-phenyl-1*H*-pyrazol-5(4*H*)-one

**DOI:** 10.1107/S1600536810054127

**Published:** 2011-01-22

**Authors:** R. Jayarajan, P. Sharmila, G. Jagadeesan, G. Vasuki, S. Aravindhan

**Affiliations:** a Department of Chemistry, Pondicherry University, Puducherry 605 014, India; b Department of Physics, Presidency College, Chennai 600 005, India

## Abstract

In the title compound C_14_H_17_N_3_O_2_, the dihedral angle between the rings is 16.68 (13)°. Although the compound crystallizes in the keto form, the possibility of keto-enamine–enol-imine tautomerism is explained by a strong intra­molecular N—H⋯O hydrogen bond.

## Related literature

4-Acyl­pyrazolo­nes are good chelating ligands and also show anti­bacterial, anti­fungal, anti-inflammatory, carcino-static and enzyme inhibitory activity, see: Patel *et al.* (2000 ▶, 2001 ▶); Chohan & Kausar (2000 ▶); Chohan, Jaffery & Supuran (2001 ▶); Chohan, Munawar & Supuran (2001 ▶); Chohan *et al.* (2002) ▶; Yang *et al.* (2000 ▶). For analgesic agents, see: Gursoy *et al.* (2000 ▶).
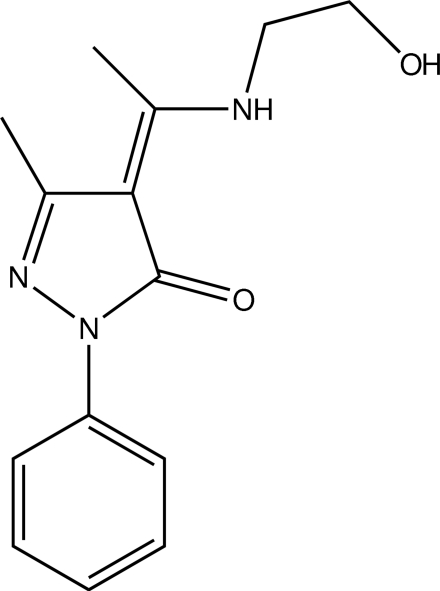


## Experimental

### 

#### Crystal data

C_14_H_17_N_3_O_2_
*M* *_r_* = 259.31
Monoclinic, 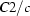

*a* = 22.4703 (13) Å
*b* = 7.0902 (4) Å
*c* = 18.0565 (11) Å
β = 110.926 (7)°
*V* = 2687.0 (3) Å^3^
*Z* = 8
Mo *K*α radiation
μ = 0.09 mm^−1^
*T* = 273 K
0.20 × 0.20 × 0.20 mm

#### Data collection

Oxford Diffraction Xcalibur Eos diffractometer
Absorption correction: multi-scan (*CrysAlis PRO*; Oxford Diffraction, 2007 ▶) *T*_min_ = 0.978, *T*_max_ = 0.982
4492 measured reflections
2353 independent reflections
1544 reflections with *I* > 2σ(*I*)
*R*_int_ = 0.026

#### Refinement

*R*[*F* ^2^ > 2σ(*F* ^2^)] = 0.055
*wR*(*F* ^2^) = 0.173
*S* = 0.98
2353 reflections
176 parameters
H atoms treated by a mixture of independent and constrained refinement
Δρ_max_ = 0.46 e Å^−3^
Δρ_min_ = −0.24 e Å^−3^

### 

Data collection: *CrysAlis PRO* (Oxford Diffraction, 2007 ▶); cell refinement: *CrysAlis PRO*; data reduction: *CrysAlis PRO*; program(s) used to solve structure: *SHELXS97* (Sheldrick, 2008 ▶); program(s) used to refine structure: *SHELXL97* (Sheldrick, 2008 ▶); molecular graphics: *ORTEP-3* (Farrugia, 1997 ▶); software used to prepare material for publication: *SHELXL97* and *PLATON* (Spek, 2009) ▶.

## Supplementary Material

Crystal structure: contains datablocks I, global. DOI: 10.1107/S1600536810054127/ds2072sup1.cif

Structure factors: contains datablocks I. DOI: 10.1107/S1600536810054127/ds2072Isup2.hkl

Additional supplementary materials:  crystallographic information; 3D view; checkCIF report

## Figures and Tables

**Table 1:** Hydrogen-bond geometry (Å, °)

*D*—H⋯*A*	*D*—H	H⋯*A*	*D*⋯*A*	*D*—H⋯*A*
N3—H⋯O1	0.90 (3)	1.92 (3)	2.711 (3)	146 (3)

**Fig. 1.**
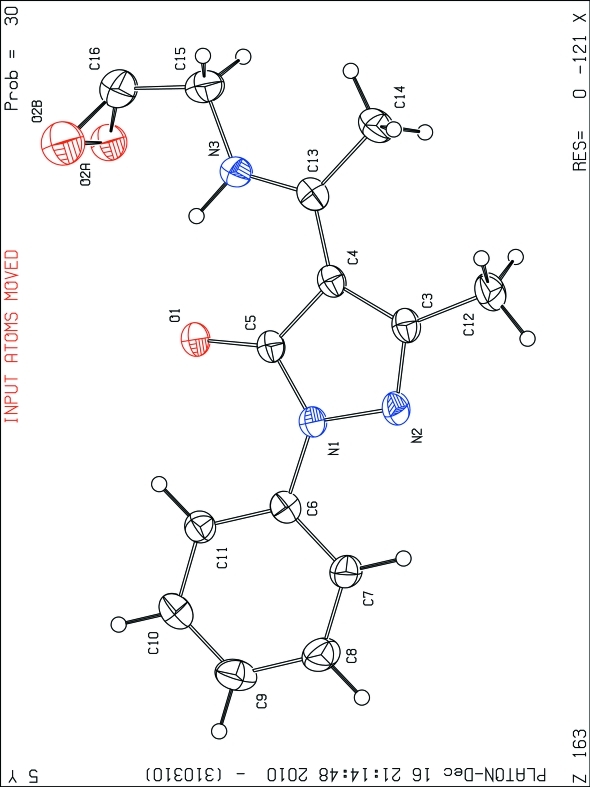
The ORTEP diagram of molecule H-atoms are involved in hydrogen bonding are shown as Dashed lines.

**Fig. 2.**
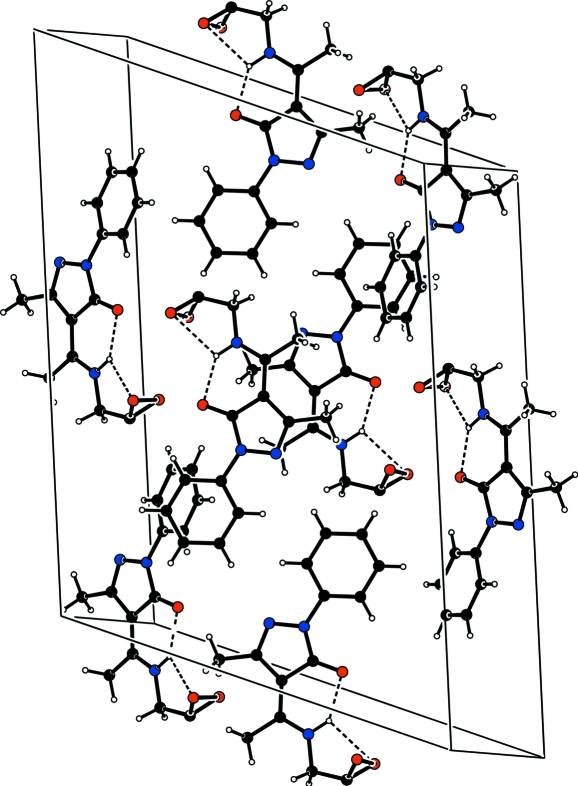
Crystal Packing Diagram.

## supplementary crystallographic information

### Comment

The *ORTEP* diagram for the molecule of the title compound is given in Fig:1. The molecule is almost planar where the phenyl ring is tilted by 17.66° to the rest of the molecule. The planarity is explained by the torsion angles C3—C4—C13—N3(-179.49°) and C13—N3—C15—C16 (173.88°).

The hydroxyl oxygen (O2) shows disorder and hence O2A and O2B were not refined using anisotropic thermal parameters.This disordered nature show a dynamic rotation about C15—C16 single bond.

The hydrogen (H3) of the imino group(N3) forms a strong intra-molecular hydrogen bond with keto oxygen O1 (2.711 Å, 146.56°). Along with this, the double bond character of C13—N3, show the possibility of proton shuttling between O1 and N3.

Though phenyl ring and pyrazol ring can adopt perpendicularity,the weak C11—H11···O1 interaction (2.954 Å, 121.13°) keeps them with near planarity.

The crystal packing diagram in Fig:2.

### Experimental

Ethanolic solution of 3-methyl-1-phenyl-4-acetylpyrazolin-5-ol (0.432 g, 2 mmol) and 2-aminoethanol (0.122 g, 2mmoL) were taken in a round bottom flask and refluxed for 4 h. The solid product was filtered and washed with cold ethanol. The product obtained was pure by TLC and NMR spectroscopy. However, the product was further purified by re-crystallization from ethanol and dried under vacuum. The compound was crystallized by slow evaporation technique using methanol as solvent at room temperature.

### Crystal data

**Table:**

C_14_H_17_N_3_O_2_	*F*(000) = 1104
*M**_r_* = 259.31	*D*_x_ = 1.282 Mg m^−^^3^
Monoclinic, *C*2/*c*	Mo *K*α radiation, λ = 0.71073 Å
Hall symbol: -C 2yc	Cell parameters from 2447 reflections
*a* = 22.4703 (13) Å	θ = 2.7–29.3°
*b* = 7.0902 (4) Å	µ = 0.09 mm^−^^1^
*c* = 18.0565 (11) Å	*T* = 273 K
β = 110.926 (7)°	Monoclinic, colourless
*V* = 2687.0 (3) Å^3^	0.20 × 0.20 × 0.20 mm
*Z* = 8	

### Data collection

**Table:**

Oxford Diffraction Xcalibur Eos diffractometer	2353 independent reflections
Radiation source: fine-focus sealed tube	1544 reflections with *I* > 2σ(*I*)
graphite	*R*_int_ = 0.026
Detector resolution: 15.9821 pixels mm^-1^	θ_max_ = 25.0°, θ_min_ = 3.0°
ω scans	*h* = −26→26
Absorption correction: multi-scan (*CrysAlis PRO*; Oxford Diffraction, 2007)	*k* = −8→4
*T*_min_ = 0.978, *T*_max_ = 0.982	*l* = −19→21
4492 measured reflections	

### Refinement

**Table:**

Refinement on *F*^2^	Primary atom site location: structure-invariant direct methods
Least-squares matrix: full	Secondary atom site location: difference Fourier map
*R*[*F*^2^ > 2σ(*F*^2^)] = 0.055	Hydrogen site location: inferred from neighbouring sites
*wR*(*F*^2^) = 0.173	H atoms treated by a mixture of independent and constrained refinement
*S* = 0.98	*w* = 1/[σ^2^(*F*_o_^2^) + (0.1157*P*)^2^] where *P* = (*F*_o_^2^ + 2*F*_c_^2^)/3
2353 reflections	(Δ/σ)_max_ = 0.001
176 parameters	Δρ_max_ = 0.46 e Å^−^^3^
0 restraints	Δρ_min_ = −0.24 e Å^−^^3^

### Special details

**Table:**

Geometry. All e.s.d.'s (except the e.s.d. in the dihedral angle between two l.s. planes) are estimated using the full covariance matrix. The cell e.s.d.'s are taken into account individually in the estimation of e.s.d.'s in distances, angles and torsion angles; correlations between e.s.d.'s in cell parameters are only used when they are defined by crystal symmetry. An approximate (isotropic) treatment of cell e.s.d.'s is used for estimating e.s.d.'s involving l.s. planes.
Refinement. Refinement of *F*^2^ against ALL reflections. The weighted *R*-factor *wR* and goodness of fit *S* are based on *F*^2^, conventional *R*-factors *R* are based on *F*, with *F* set to zero for negative *F*^2^. The threshold expression of *F*^2^ > σ(*F*^2^) is used only for calculating *R*-factors(gt) *etc*. and is not relevant to the choice of reflections for refinement. *R*-factors based on *F*^2^ are statistically about twice as large as those based on *F*, and *R*- factors based on ALL data will be even larger.

### Fractional atomic coordinates and isotropic or equivalent isotropic displacement parameters (Å^2^)

**Table:**

	*x*	*y*	*z*	*U*_iso_*/*U*_eq_	Occ. (<1)
O1	−0.05676 (8)	0.3364 (3)	0.35564 (9)	0.0534 (5)	
N1	−0.11378 (9)	0.2982 (3)	0.44012 (10)	0.0384 (5)	
N2	−0.10103 (10)	0.2539 (3)	0.51998 (10)	0.0439 (5)	
C11	−0.19444 (10)	0.4030 (3)	0.31598 (12)	0.0432 (6)	
H11	−0.1628	0.4457	0.2982	0.052*	
C10	−0.25819 (11)	0.4237 (4)	0.26886 (14)	0.0523 (7)	
H10	−0.2690	0.4794	0.2192	0.063*	
N3	0.06883 (10)	0.2770 (3)	0.43754 (13)	0.0500 (6)	
C6	−0.17841 (10)	0.3190 (3)	0.38911 (12)	0.0378 (6)	
C4	−0.00882 (11)	0.2571 (3)	0.49473 (13)	0.0382 (6)	
C9	−0.30523 (12)	0.3635 (4)	0.29449 (15)	0.0586 (8)	
H9	−0.3478	0.3793	0.2628	0.070*	
C13	0.05492 (11)	0.2441 (3)	0.50051 (13)	0.0397 (6)	
C14	0.10798 (12)	0.1939 (4)	0.57534 (14)	0.0539 (7)	
H14A	0.1475	0.1930	0.5661	0.081*	
H14B	0.1004	0.0711	0.5926	0.081*	
H14C	0.1103	0.2852	0.6155	0.081*	
C8	−0.28911 (13)	0.2791 (4)	0.36768 (16)	0.0627 (8)	
H8	−0.3210	0.2374	0.3852	0.075*	
C3	−0.03936 (12)	0.2315 (3)	0.55172 (13)	0.0413 (6)	
C7	−0.22638 (12)	0.2558 (4)	0.41503 (14)	0.0510 (7)	
H7	−0.2159	0.1980	0.4643	0.061*	
C5	−0.05950 (11)	0.3010 (3)	0.42224 (12)	0.0379 (6)	
C12	−0.01114 (13)	0.1893 (4)	0.63896 (13)	0.0569 (7)	
H12A	−0.0446	0.1807	0.6602	0.085*	
H12B	0.0177	0.2884	0.6654	0.085*	
H12C	0.0115	0.0718	0.6470	0.085*	
C16	0.12548 (15)	0.2950 (5)	0.34639 (18)	0.0694 (9)	
C15	0.13080 (13)	0.2678 (5)	0.42954 (18)	0.0677 (8)	
H15A	0.1501	0.1461	0.4481	0.081*	
H15B	0.1583	0.3644	0.4624	0.081*	
O2A	0.08008 (17)	0.1945 (5)	0.2936 (2)	0.0683 (13)*	0.567 (5)
O2B	0.0870 (2)	0.4236 (7)	0.3041 (3)	0.0637 (16)*	0.433 (5)
H	0.0343 (14)	0.315 (4)	0.3970 (16)	0.063 (8)*	

### Atomic displacement parameters (Å^2^)

**Table:**

	*U*^11^	*U*^22^	*U*^33^	*U*^12^	*U*^13^	*U*^23^
O1	0.0487 (10)	0.0768 (13)	0.0344 (9)	0.0027 (9)	0.0143 (8)	0.0094 (8)
N1	0.0392 (11)	0.0452 (11)	0.0299 (10)	0.0004 (8)	0.0115 (8)	0.0011 (8)
N2	0.0461 (12)	0.0548 (13)	0.0294 (10)	−0.0018 (9)	0.0116 (9)	0.0022 (9)
C11	0.0442 (13)	0.0431 (13)	0.0410 (13)	−0.0009 (11)	0.0135 (11)	−0.0001 (10)
C10	0.0544 (15)	0.0531 (15)	0.0421 (14)	0.0072 (12)	0.0084 (12)	0.0011 (12)
N3	0.0375 (12)	0.0651 (15)	0.0450 (13)	0.0036 (10)	0.0120 (10)	0.0048 (11)
C6	0.0441 (13)	0.0344 (12)	0.0336 (12)	0.0012 (10)	0.0122 (10)	−0.0046 (10)
C4	0.0437 (13)	0.0346 (12)	0.0323 (12)	−0.0009 (10)	0.0087 (10)	0.0005 (10)
C9	0.0436 (14)	0.077 (2)	0.0485 (16)	0.0075 (13)	0.0083 (12)	−0.0075 (14)
C13	0.0467 (14)	0.0324 (12)	0.0356 (13)	−0.0027 (10)	0.0093 (11)	−0.0034 (10)
C14	0.0487 (15)	0.0547 (15)	0.0471 (15)	−0.0001 (12)	0.0035 (12)	−0.0006 (12)
C8	0.0467 (16)	0.092 (2)	0.0538 (17)	−0.0024 (14)	0.0234 (13)	−0.0078 (15)
C3	0.0489 (14)	0.0369 (13)	0.0350 (12)	−0.0027 (10)	0.0110 (11)	−0.0003 (10)
C7	0.0445 (14)	0.0710 (19)	0.0388 (14)	−0.0002 (13)	0.0163 (12)	−0.0012 (12)
C5	0.0443 (13)	0.0381 (12)	0.0310 (12)	−0.0007 (10)	0.0131 (10)	0.0014 (9)
C12	0.0632 (17)	0.0698 (18)	0.0328 (13)	−0.0007 (14)	0.0110 (12)	0.0068 (12)
C16	0.0639 (18)	0.091 (2)	0.0568 (18)	−0.0034 (17)	0.0262 (15)	0.0007 (17)
C15	0.0434 (16)	0.094 (2)	0.0663 (19)	0.0048 (14)	0.0208 (14)	0.0060 (16)

### Geometric parameters (Å, °)

**Table:**

O1—C5	1.251 (3)	C9—H9	0.9300
N1—C5	1.369 (3)	C13—C14	1.491 (3)
N1—N2	1.402 (2)	C14—H14A	0.9600
N1—C6	1.423 (3)	C14—H14B	0.9600
N2—C3	1.306 (3)	C14—H14C	0.9600
C11—C6	1.374 (3)	C8—C7	1.373 (3)
C11—C10	1.388 (3)	C8—H8	0.9300
C11—H11	0.9300	C3—C12	1.503 (3)
C10—C9	1.365 (4)	C7—H7	0.9300
C10—H10	0.9300	C12—H12A	0.9600
N3—C13	1.303 (3)	C12—H12B	0.9600
N3—C15	1.452 (4)	C12—H12C	0.9600
N3—H	0.90 (3)	C16—O2B	1.300 (5)
C6—C7	1.394 (4)	C16—O2A	1.328 (4)
C4—C13	1.401 (4)	C16—C15	1.476 (4)
C4—C3	1.438 (3)	C15—H15A	0.9700
C4—C5	1.429 (3)	C15—H15B	0.9700
C9—C8	1.376 (4)		
			
C5—N1—N2	111.97 (17)	H14A—C14—H14C	109.5
C5—N1—C6	129.44 (18)	H14B—C14—H14C	109.5
N2—N1—C6	118.40 (18)	C7—C8—C9	120.7 (3)
C3—N2—N1	105.88 (18)	C7—C8—H8	119.7
C6—C11—C10	119.6 (2)	C9—C8—H8	119.7
C6—C11—H11	120.2	N2—C3—C4	111.9 (2)
C10—C11—H11	120.2	N2—C3—C12	117.9 (2)
C9—C10—C11	120.9 (2)	C4—C3—C12	130.1 (2)
C9—C10—H10	119.5	C8—C7—C6	119.8 (2)
C11—C10—H10	119.5	C8—C7—H7	120.1
C13—N3—C15	128.0 (2)	C6—C7—H7	120.1
C13—N3—H	111.0 (18)	O1—C5—N1	125.7 (2)
C15—N3—H	120.9 (18)	O1—C5—C4	128.8 (2)
C11—C6—C7	119.6 (2)	N1—C5—C4	105.45 (19)
C11—C6—N1	121.6 (2)	C3—C12—H12A	109.5
C7—C6—N1	118.8 (2)	C3—C12—H12B	109.5
C13—C4—C3	132.4 (2)	H12A—C12—H12B	109.5
C13—C4—C5	122.8 (2)	C3—C12—H12C	109.5
C3—C4—C5	104.8 (2)	H12A—C12—H12C	109.5
C10—C9—C8	119.4 (2)	H12B—C12—H12C	109.5
C10—C9—H9	120.3	O2B—C16—O2A	77.0 (3)
C8—C9—H9	120.3	O2B—C16—C15	119.0 (3)
N3—C13—C4	118.7 (2)	O2A—C16—C15	114.9 (3)
N3—C13—C14	118.0 (2)	N3—C15—C16	111.3 (2)
C4—C13—C14	123.3 (2)	N3—C15—H15A	109.4
C13—C14—H14A	109.5	C16—C15—H15A	109.4
C13—C14—H14B	109.5	N3—C15—H15B	109.4
H14A—C14—H14B	109.5	C16—C15—H15B	109.4
C13—C14—H14C	109.5	H15A—C15—H15B	108.0

### Hydrogen-bond geometry (Å, °)

**Table:**

*D*—H···*A*	*D*—H	H···*A*	*D*···*A*	*D*—H···*A*
N3—H···O1	0.90 (3)	1.92 (3)	2.711 (3)	146 (3)
